# Polychlorinated Biphenyl-126 Activates AXL/ERb/DNMT3A Axis to Drive Endometriosis Progression

**DOI:** 10.21203/rs.3.rs-6631264/v1

**Published:** 2025-05-13

**Authors:** Yuri Park, Nuri Sung, Eunsu Kim, Jaeyeong Jeong, Juhee Sim, Mi Jin Park, John Lydon, Xiaoming Guan, Sang Jun Han

**Affiliations:** Baylor College of Medicine; Baylor College of Medicine; Baylor College of Medicine; Baylor College of Medicine; Baylor College of Medicine; Baylor College of Medicine; Baylor College of Medicine; Baylor College of Medicine; Baylor College of Medicine

**Keywords:** endometriosis, PCB126, Estrogen Receptor beta, AXL, DNMT3A

## Abstract

Endometriosis is a pathological condition in which endometrial cells proliferate outside the uterine cavity, resulting in pelvic pain and infertility. Exposure to endocrine-disrupting chemicals (EDCs) has been implicated in the progression of endometriosis, though the precise mechanisms remain largely undefined. Among EDCs, elevated levels of polychlorinated biphenyl (PCB)-126 have been strongly associated with endometriosis, particularly in patients with deep infiltrating disease. In a mouse model of endometriosis, PCB-126 exposure significantly promoted the growth of ectopic lesions by activating the Steroid Receptor Coactivator-1 (SRC-1) isoform/Matrix Metalloproteinase-9 (MMP9)/Estrogen Receptor-β (ERβ) axis, a key driver of disease progression. PCB-126 also enhanced ERβ activity via upregulation of the AXL Receptor Tyrosine Kinase (AXL)/Growth Arrest-Specific 6 (GAS6) signaling pathway in endometriotic lesions. Notably, BMS-777607, an AXL inhibitor, effectively suppressed lesion growth in this model. Moreover, the PCB-126/ERβ axis directly increased expression of DNA Methyltransferase 3A (DNMT3A), contributing to inflammation and immune dysregulation in endometriotic tissue. Collectively, these findings suggest that PCB-126 promotes endometriosis progression through coordinated activation of the AXL/ERβ/DNMT3A axis, driving estrogen-mediated epigenetic and immunoinflammatory responses.

## Introduction

Endometriosis is a medical condition in which endometrial cells grow outside the uterine cavity, affecting up to 10% of women of reproductive age and causing symptoms such as infertility and severe pelvic pain^1^. Due to the severe chronic morbidities associated with endometriosis, numerous studies have sought to identify the distinct molecular features of endometriotic lesions in order to develop more effective prognostic, diagnostic, and therapeutic strategies.

Several hypotheses have been proposed to explain the initiation and progression of endometriosis in approximately 10% of women of reproductive age^2^. Among these, exposure to environmental endocrine disruptors (EDRs)—such as polychlorinated biphenyls (PCBs), dioxins, bisphenol A, and phthalates—has been identified as a potential contributing factor in the progression of endometriosis^3^. For example, women exposed to moderate levels of PCBs (5–8 parts per billion [ppb]) or high levels (> 8 ppb) have a higher incidence of endometriosis compared to those with low PCB exposure (< 5 ppb)^4^. In addition, elevated serum levels of dioxin-like PCB congeners have been detected in Italian women with endometriosis compared to healthy controls^5^. A study conducted in Belgium, Italy, and India found elevated total PCB concentrations in patients with adenomyosis and peritoneal endometriosis compared to controls^6^. The primary route of human exposure to PCBs is through the consumption of contaminated food^7, 8^. Thus, accumulating evidence supports the hypothesis that women with high levels of PCB exposure are at increased risk of developing endometriosis compared to those with lower exposure levels.

Among PCBs, we are particularly interested in 3,3′,4,4′,5-pentachlorobiphenyl (PCB126) due to its unique molecular properties associated with endometriosis. PCB126 is considered the most potent dioxin-like PCB and has the highest Toxic Equivalency Factor (TEF) among PCBs, at 0.1—second only to TCDD, which has a TEF of 1.0. Owing to its high potency, PCB126 is often the primary contributor to the overall toxic equivalency in common PCB mixtures, accounting for up to 90% of the total dioxin-like activity^9^. Elevated levels of PCB126 have been detected in the adipose tissue of patients with deep infiltrating endometriosis^8^. PCB126 exposure stimulates the mRNA expression of steroidogenic genes—such as Steroidogenic Acute Regulatory Protein (STAR), Hydroxy-Delta-5-Steroid Dehydrogenase 3 Beta (HSD3B), and Cytochrome P450 Family 19 Subfamily A Member 1 (CYP19A1)—in chicken ovarian follicles, leading to elevated local estrogen levels, a key feature of endometriosis^10, 11^.

PCB126 exhibits estrogenic activity in certain contexts, although its effects are complex. For example, PCB126 enhances estradiol (E₂) production in fish ovarian follicles, mimicking the effects of exogenous E₂ by stimulating aromatase activity and modulating steroidogenic enzyme pathways^12^. Additionally, PCB126 induces estrogen receptor (ER)-responsive genes alongside aryl hydrocarbon receptor (AhR)-mediated responses^13^. This study revealed that PCB126 may promote estrogen-dependent disease progression, such as endometriosis, by facilitating crosstalk between AhR signaling and ER pathways.

PCB126 exposure induces dynamic changes in epigenetic regulation, including DNA methylation, histone modifications, and RNA methylation, in a tissue-dependent manner. Prenatal exposure to PCB126 in mice upregulates Dnmt1 and Dnmt3b expression in the fetal liver, resulting in global DNA hypermethylation^14^. Exposure to PCB126 also upregulates histone lysine methyltransferases, such as kmt2a and setd1a, in zebrafish, leading to altered chromatin accessibility^15^. Alterations in epigenetic regulation are significantly associated with endometriosis progression. For example, dysregulated expression of DNA methyltransferases (DNMT1, DNMT3A, and DNMT3B) in endometriotic lesions, along with differential DNA methylation patterns in genes involved in inflammatory pathways, steroid signaling, and apoptosis, have been linked to the development and progression of endometriosis^16, 17^.

The above observations strongly suggest that exposure to PCB126 may be a causal factor in the initiation and progression of endometriosis. However, the precise mechanisms by which PCB126 promotes endometriosis progression remain unclear.

## Material and Methods

### Rigorous Experimental Design

All investigators will be blinded to mouse genotype information.

#### Animal numbers and power calculations.

The required minimal number of animals per group was determined using a power calculation to ensure adequate statistical power (α = 0.05, power = 80%) to detect a biologically significant difference. Sample size estimation was performed using GPower^18^ based on each experiment data.

#### Mice.

C57BL/6J female mice (6 weeks old) and Severe Combined Immunodeficiency (SCID) female mice (6 weeks old) were purchased from Jackson Laboratory. C57BL/6J, SCID, ROSA^LSL:ERβ/+^ :PR^Cre/+19^, and PR^Cre/+20^, Dnmt3a^f/f21^, and Dnmt3a^f/f^:PR^Cre/+^ mice were maintained in the designated animal care facility at Baylor College of Medicine according to the Institutional Animal Care and Use Committee (IACUC) guidelines for the care and use of laboratory animals. An IACUC-approved protocol was followed for all animal experiments in this study. The assurance number of our animal protocol is D16–00475.

#### Immortalized human endometrial cells.

Immortalized human endometrial stromal cells (IHESCs)^22^ and EMosis-CC/TERT1 (immortalized human endometriotic epithelial cells)^23^, ERβ overexpressing IHEESCs^19^ were employed and confirmed by Short Tandem Repeat profiling; these cells were not contaminated with mycoplasma.

#### PCB126 effect on SRC-1 isoform, ERβ, and MMP9 in IHEECs.

IHEECs were treated with 0.1 nM PCB126 or vehicle control for 24 hours in DMEM/F12 medium supplemented with 10% FBS, penicillin (100 U/mL), streptomycin (100 μg/mL), and amphotericin B (2.5 μg/mL) under humidified conditions (5% CO₂, 95% air) at 37°C. Following treatment, total protein lysates were prepared from PCB126- and vehicle-treated IHEECs. The levels of SRC-1 isoform, ERβ, and MMP9 were assessed by Western blot analysis. Tubulin was used as a loading control.

#### 3-(4,5-Dimethylthiazol-2-yl)-5-(3-carboxymethoxyphenyl)-2-(4-sulfophenyl)-2H-tetrazolium) (MTS) cell growth assay.

Human endometrial cells were seeded into 96-well plates at a density of 1 × 10⁴ cells per well. The following day, cells were treated with serial dilutions of PCB126. After 3 days of treatment, 10μL of MTS reagent (Promega, catalog number: G1111) was added to each well, and the plates were incubated for 2 hours. Optical density was then measured at 450 nm using a microplate reader.

#### Surgically induced endometriosis (Autotransplantation):

Endometriosis was surgically induced in mice under anesthesia and aseptic conditions using a modified method described previously^24^. Briefly, C57BL/6J female mice (6 weeks old) were implanted subcutaneously with a sterile 60-day release pellet containing 0.36 mg of 17-β estradiol (Innovative Research of America). Two days after the implantation, one uterine horn from each mouse was isolated under anesthesia. In a Petri dish containing pre-warmed DMEM/F-12 medium (Invitrogen) supplemented with 100 U/mL penicillin and 100 μg/mL streptomycin, the uterine horns were longitudinally incised with scissors. A 2-mm endometrial fragment was then excised using a dermal biopsy punch (Miltex) and sutured to the mesenteric membrane attached to the intestine of the same mouse through a midline abdominal incision, using a 7 − 0 braided polypropylene suture (Ethicon). For endometriosis control mice, the suture was performed without endometrial tissue implantation. The abdominal incision was closed using a 5 − 0 braided polypropylene suture (Ethicon) in a continuous manner. On day 21 post-surgery, mice were euthanized, and both endometriotic lesions and eutopic endometria were carefully dissected from the surrounding tissues. In control mice, the uterus was collected 21 days after the sham endometriosis procedure. The volume of endometriotic lesions was calculated using the following formula: Volume (mm³) = 0.52 × width × length × height.

#### Endometriosis by heterotransplantation with cultured human endometrial cells:

For noninvasive analysis of ectopic lesion growth in Severe Combined Immunodeficiency (SCID) mice, luciferase-labeled IHEECs (Luc-IHEECs) and luciferase-labeled IHESCs (Luc-IHESCs) were used^19^. Cells were cultured in DMEM/F12 medium supplemented with 10% FBS, penicillin (100 U/mL), streptomycin (100 μg/mL), and amphotericin B (2.5 μg/mL) under humidified conditions (5% CO₂, 95% air) at 37°C. The culture medium was replaced every other day. On the day of transplantation, cells were trypsinized using 0.1% trypsinEDTA. Luc-IHESCs (2 × 10⁶ cells) were mixed with Luc-IHEECs (2 × 10⁶ cells) in 10 mL of DMEM/F12, pelleted, and washed. The mixed cell pellet was resuspended in 100 μL of DMEM/F12 and combined with 100 μL of Matrigel (BD Biosciences) at a 1:1 ratio. A total of 200 μL of the cell-Matrigel suspension was injected intraperitoneally along the midventral line just caudal to the umbilicus of SCID female mice (6 weeks old) previously implanted with a sterile 60-day release pellet containing 0.36 mg of 17-β estradiol (Innovative Research of America, Florida, USA).

#### Endometriosis to generate Dnmt3a knockout (KO) ectopic lesions.

Dnmt3^f/f^:PR^Cre/+^ and Dnmt3a^f/f^ female mice (n = 4 per group, 8 weeks old) were subcutaneously injected with 17β-estradiol (E2) at a dose of 100 ng per 20 g of body weight once daily for 3 consecutive days. After treatment, uterine horns from each mouse were isolated under anesthesia and longitudinally incised with scissors. A 2-mm endometrial fragment was then obtained using a dermal biopsy punch. Syngeneic recipient C57BL/6J female mice (8 weeks old, n = 8) were implanted subcutaneously with a sterile, 60-day release pellet containing 0.36 mg of 17β-estradiol (Innovative Research of America) two days prior to endometriosis surgery. On the day of surgery, one endometrial fragment from a donor mouse was sutured to the mesenteric membrane of a recipient mouse through a midline abdominal incision using a 7 − 0 braided polypropylene suture (Ethicon). The incision was then closed with a 5 − 0 braided polypropylene suture (Ethicon) in a continuous pattern. On day 21 following endometriosis induction, mice were euthanized, and endometriotic lesions as well as eutopic endometria were carefully dissected from surrounding tissues. Lesion volume was calculated using the formula: Volume (mm³) = 0.52 × width × length × height. The minimum number of animals required for endometriosis methods to assess the effect of Dnmt3a KO on endometriosis, achieving 80% power with a p-value of 0.05, is one mouse.

#### PCB126 exposure during endometriosis progression.

After endometriosis was induced in mice using both auto- and heterotransplantation methods, the mice were randomly divided into two groups. One group received intraperitoneal injections of PCB126 at a dose of 1 mg/kg once per week for 5 weeks. The control group received intraperitoneal injections of vehicles alone on the same schedule. The minimum number of animals required for both auto- and heterotransplantation methods to assess the effect of PCB126 on endometriosis, achieving 80% power with a p-value of 0.05, is two mice.

#### BMS-777607 inhibit endometriosis progression.

After endometriosis was induced in mice using autotransplantation, the mice were randomly divided into two groups. One group received intraperitoneal injections of BMS-777607 (25 mg/kg; Selleckchem, catalog number: S1562) five times per week for 3 weeks. The control group received intraperitoneal injections of vehicles on the same schedule. The minimum number of animals required for both auto transplantation methods to assess the effect of BMS-777607 on endometriosis, achieving 90% power with a p-value of 0.01, is one mouse.

#### Mouse Receptor Tyrosine Kinase (RTK) Array Analysis.

To assess differential receptor tyrosine kinase (RTK) activation between normal and endometriotic tissues, as well as between ectopic lesions exposed to PCB126 or vehicle, the Mouse Phospho-RTK Array Kit (R&D Systems, catalog number: ARY014) was used according to the manufacturer’s instructions. Briefly, normal endometrial tissues, endometriotic lesions, and ectopic lesions treated with PCB126 or vehicle were freshly harvested from mice. Total cell lysates were prepared from the isolated tissues. Equal amounts of protein (300 μg per array membrane) from each sample group were incubated with pre-blocked phospho-RTK array membranes overnight at 4°C on a rocking platform. After washing to remove unbound proteins, the membranes were incubated with a cocktail of biotinylated anti-phosphotyrosine detection antibodies, followed by a streptavidinhorseradish peroxidase (HRP) conjugate. Chemiluminescent signals were developed using the supplied substrate and visualized with a chemiluminescent imaging system. Dot intensities corresponding to phosphorylated RTKs were quantified using ImageJ software, normalized to internal reference spots, and compared across sample groups to identify differentially activated RTKs.

#### Western Blot Analyses.

Primary antibodies against the following proteins were used: SRC-1 (ab10308; Abcam, 1:1000 dilution), ERβ antibody (Invitrogen, catalog number: PPZ0506), MMP9 (ab38898; Abcam, 1:1000 dilution), and tubulin (antibody information to be specified). Membranes were incubated with HRP-conjugated secondary antibodies (Sigma), and signals were detected using the SuperSignal^™^ West Femto Chemiluminescent Substrate (Thermo Scientific).

#### Imaging and Quantification of Bioluminescence Data:

Mice were anesthetized with 1.5% isoflurane in air using an inhalation anesthesia system (VetEquip). D-Luciferin (Xenogen) was administered via intraperitoneal injection at a dose of 40 mg/kg body weight. Ten minutes after injection, mice were imaged using an IVIS Imaging System (Xenogen) under continuous isoflurane anesthesia (1–2%). Imaging parameters were kept consistent across all sessions for comparative analysis. Grayscale reference images were superimposed with pseudocolor images representing bioluminescence signals and analyzed using Living Image software (Version 4.4, Xenogen). A region of interest (ROI) was manually drawn over the area of signal intensity, and the ROI size was kept constant across all samples. Signal intensity was quantified as total photon flux (photons/sec/cm²) within the ROI.

#### PCB126 effect on ERα and ERβ activity.

HeLa cells were cultured in phenol red–free DMEM supplemented with 10% charcoal-stripped serum. Cells were then plated into 24-well plates. Once they reached approximately 90% confluence, HeLa cells were transiently transfected using Lipofectamine 2000 (Thermo Fisher, catalog number: [insert reference]) with an ERβ expression vector in the pCR3.1 backbone (5 ng/well) and a luciferase reporter plasmid containing five copies of the Estrogen Response Element (ERE; 400 ng/well). As a control for ERβ, a parallel group of HeLa cells was transfected with an ERα expression vector in the same vector backbone (5 ng/well) along with the same ERE-luciferase reporter (400 ng/well). Two days of post-transfection, cells were treated with PCB126 at concentrations ranging from 0 to 200 nM for an additional 2 days. As a positive control, a separate set of cells was treated with 10 nM estradiol for 2 days. Luciferase activity was measured using the Luciferase Assay System (Promega, catalog number: E1500) according to the manufacturer's instructions.

#### PCB126 Effects on GAS6 and ESR2 Expression Levels Using RT-qPCR

IHEECs and IHESCs were cultured as previously described. To assess whether PCB126 increases GAS6 and ESR2 expression levels, cells were seeded in 6-well plates one day prior to treatment. Once the cells reached approximately 80% confluency, various concentrations of PCB126, diluted in DMSO, were added. After 24 hours of treatment, total RNA was isolated using the RNeasy Plus Mini Kit (Qiagen, catalog number: 74134). First-strand cDNA was synthesized from 1 μg of total RNA using the SuperScript II Reverse Transcriptase Kit (Invitrogen, catalog number: 18064022) according to the manufacturer’s instructions. Gene expression levels of GAS6 and ESR2 were quantified using TaqMan probes for GAS6 (Invitrogen, catalog number: Hs01090305_m1) and ESR2 (Invitrogen, catalog number: Hs00230957_m1). Relative mRNA expression was calculated using the 2^−ΔΔCT method and normalized to 18S rRNA levels.

#### Effect of GAS6 on ERβ Transcriptional Activity Using the ERE Luciferase Assay

HeLa cells were seeded in 24-well plates and cultured in phenol red–free DMEM supplemented with 10% charcoal-stripped serum. When cells reached approximately 80% confluency, they were transiently transfected with 500 ng of the pCR3.1-ERE-luc reporter plasmid and 50 ng of the ERβ expression plasmid (pCR3.1-ERβ) using Lipofectamine 2000 (ThermoFisher, catalog number: 116680300), following the manufacturer’s protocol. Three days after transfection, cells were treated with varying concentrations (0–100 ng/mL) of GAS6 protein (R&D Systems, catalog number: 885-GSB). After 48 hours of treatment, relative luciferase activity was measured using the Luciferase Assay System (Promega, catalog number: E1500).

#### RNA-sequencing analysis of Dnmt3a ectopic lesions

Dnmt3a knockout (KO) ectopic lesions (n = 3) and wild-type (WT) control lesions (n = 3) were generated using Dnmt3a^f/f^ :PR^Cre/+^ (8 weeks old, n = 3) and Dnmt3a^f/f^ (8 weeks old, n = 3) female donor mice, as described above. Total RNA was extracted from Dnmt3a KO and control ectopic lesions using the RNeasy Plus Mini Kit (Qiagen, catalog number: 74134), according to the manufacturer’s instructions. To minimize genomic DNA contamination, the spin column membrane was additionally treated with DNase I (2 U/μL). RNA quality was assessed using a NanoDrop spectrophotometer, Invitrogen Qubit 2.0 fluorometric quantitation assay, and Agilent Bioanalyzer. RNA libraries were prepared using the Illumina TruSeq Stranded mRNA Library Preparation Kit. Sequence reads were trimmed to remove adapter sequences and low-quality bases using Galaxy version 23.1.rc1^25^. Trimmed sequence reads were mapped to the GRCm38 reference genome. Read count extraction and normalization were subsequently performed using tools on the Galaxy platform. The processed data was visualized as a heatmap using the heatmap generation tool in Galaxy version 23.1.rc1.

#### Formalin-Fixed Paraffin-Embedded (FFPE) for Human Endometriotic Lesions and

##### Normal Endometrium

Ovarian endometriomas were obtained from patients with endometriosis during surgical procedures at Baylor College of Medicine under an Institutional Review Board (IRB)-approved protocol. Normal endometrial tissue was collected from uteri removed during hysterectomies performed for uterine fibroids, also under an IRB-approved protocol at Baylor College of Medicine. All patients had discontinued exogenous hormonal treatments for at least three months prior to surgery. Both endometriotic lesions and normal endometrial tissues were fixed in 10% buffered formalin phosphate for 24 hours and subsequently stored in 70% ethanol. The tissues were dehydrated using ethanol and xylene in a tissue processor and then embedded in paraffin.

##### Gene Expression Omnibus (GEO)

RNA expression profiles comparing control and Dnmt3a KO ectopic lesions in mice with endometriosis have been deposited in the GEO database under accession number GSE296259.

#### Statistical analyses

Statistical analyses were performed using GraphPad Prism 8. P values < 0.05 were considered statistically significant.

### Results

#### The PCB126 exposure elevates endometriosis progression.

**Figure 1:**
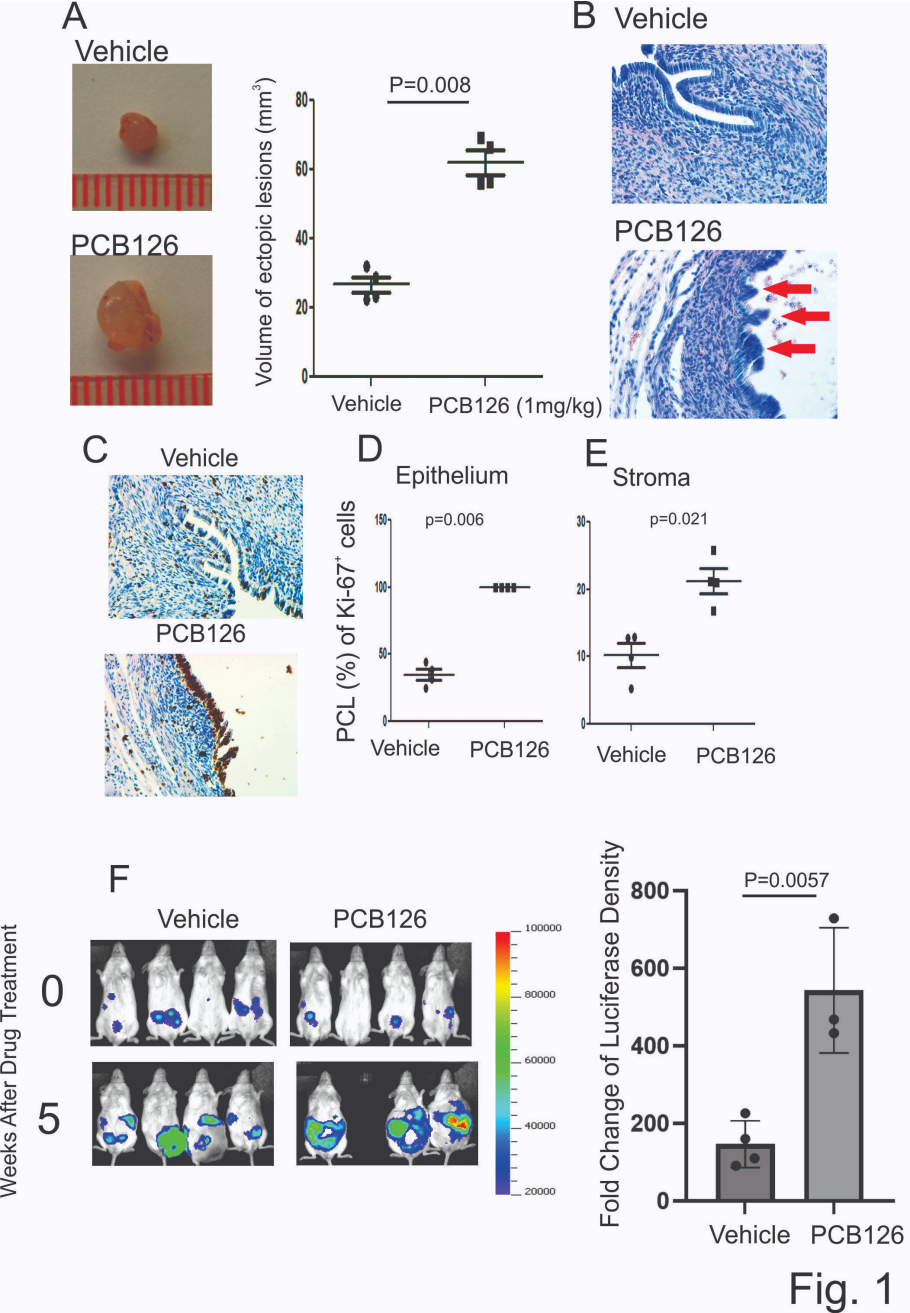
PCB126 enhanced endometriosis progression in mice with endometriosis. **A** Volume analysis of ectopic lesions isolated from mice with endometriosis treated with PCB126 (1 mg/kg) or vehicle, administered once weekly for 5 weeks. **B** Hematoxylin and Eosin (H&E) staining of ectopic lesions from vehicle- and PCB126-treated mice shown in panel A. **C** Immunohistochemical analysis of Ki-67 expression in ectopic lesions treated with vehicle or PCB126. **D–E** Quantification of the percentage of Ki-67–positive cells (PCL) in the epithelial (**D**) and stromal (**E**) compartments of ectopic lesions from vehicle- and PCB126-treated groups. **F** Evaluation of PCB126-induced progression of human ectopic lesions in SCID mice. Luciferase activity was imaged using IVIS before and after 5 weeks of treatment with PCB126 (1 mg/kg) or vehicle. Luciferase fold change was calculated as the ratio of post- to pre-treatment signal.

Elevated levels of PCB126 are correlated with the progression of endometriosis in women^26^. However, there is no direct evidence demonstrating whether and how PCB126 exposure promotes the progression of endometriosis. To address this critical question, endometriosis was surgically induced in female mice using the autotransplantation method, followed by intraperitoneal injection of PCB126 (1 mg/kg) or vehicle as a control. Compared to vehicle-treated mice, PCB126 exposure significantly increased the volume of ectopic lesions (Fig. 1A). In addition to lesion enlargement, H&E staining revealed that PCB126 induced multilayered epithelial structures within the endometriotic lesions, a feature not observed in the vehicle-treated group (Fig. 1B).

Furthermore, immunohistochemistry (IHC) for Ki-67, a proliferation marker, showed significantly increased cellular proliferation in both epithelial and stromal compartments of the lesions in PCB126-treated mice compared to controls (Figs. 1C, 1D, and 1E). These findings indicate that PCB126 exposure enhances the proliferation of epithelial and stromal cells, promoting an atypical and more aggressive form of endometriosis in this murine model.

The above observations also raised the question of whether PCB126 exposure promotes the progression of human endometriosis. To investigate this, we injected a mixture of immortalized human endometrial epithelial cells (IHEECs) and stromal cells (IHESCs) into Severe Combined Immunodeficiency (SCID) mice to generate human endometriotic lesions using a heterotransplantation method, as described in a previous study^19^. To non-invasively monitor the growth of human endometriotic lesions in mice, luciferase-labeled immortalized human endometrial epithelial and stromal cells were used^19^. Comparative analysis of *in vivo* luciferase imaging revealed that PCB126 exposure significantly increased luciferase activity in human endometriotic lesions in SCID mice compared to vehicle-treated controls (Fig. 1F). These findings indicate that PCB126 exposure promotes the progression of both murine and human endometriotic lesions in mouse models.

#### The exposure of PCB126 elevated the endometriosis driver in endometriotic lesions.

**Figure 2:**
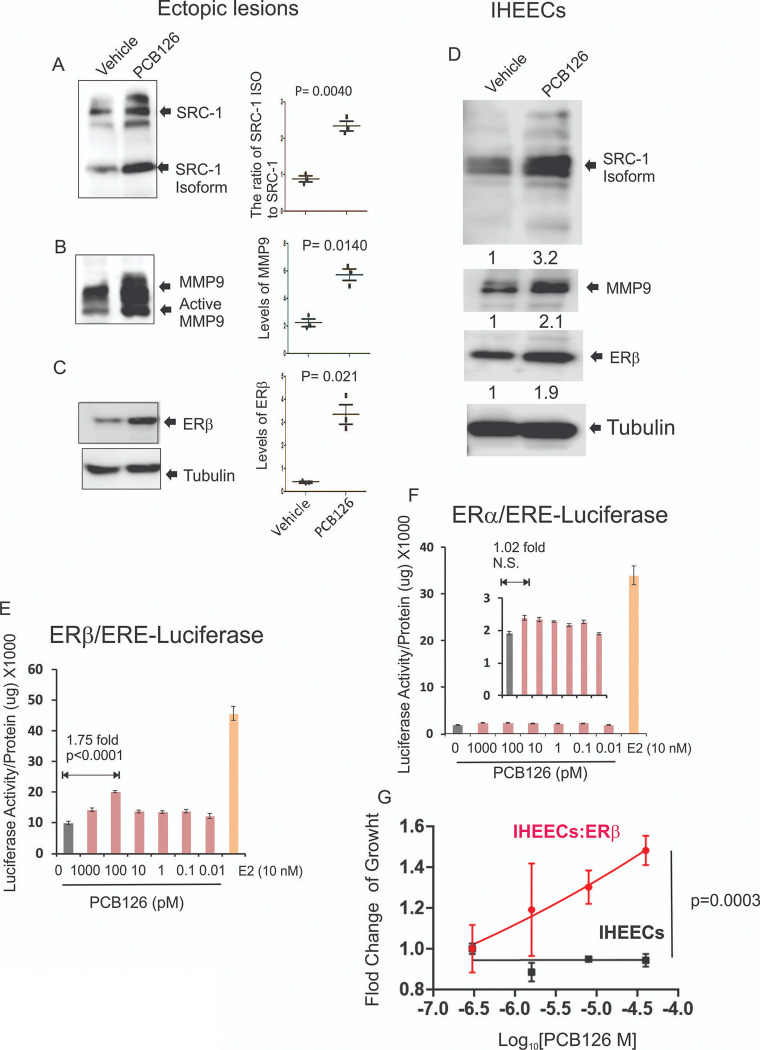
PCB126 elevated the SRC-1 isoform/MMP9/ERb axis in endometriotic lesions. **A–C** Western blot analysis to assess levels of SRC-1 and its isoform (**A**), MMP9 (**B**), and ERβ (**C**) in ectopic lesions isolated from mice with endometriosis treated with PCB126 (1 mg/kg) or vehicle once weekly for 5 weeks. Tubulin was used as a loading control for normalization. **D** Western blot analysis of SRC-1 isoform, MMP9, and ERβ in IHEECs treated with 0.1 nM PCB126 or vehicle for 2 days. Tubulin was used for normalization. **E–F** Luciferase reporter assay to evaluate the effect of PCB126 on the intrinsic transcriptional activity of ERβ (**E**) and ERα (**F**) in HeLa cells. Cells were transiently transfected with ERβ or ERα expression vectors along with an ERE-luciferase reporter construct, followed by treatment with varying doses of PCB126 or vehicle. As a positive control for ER activation, cells were treated with 10 nM E2. **G**Assessment of cell proliferation in IHEECs expressing ERβ versus parental IHEECs after treatment with increasing concentrations of PCB126 for 3 days. Cell growth was measured using the MTS assay, and fold change was calculated as the ratio of cell growth under each treatment condition relative to the vehicle control.

The Steroid Receptor Coactivator-1 (SRC-1) isoform/Matrix Metalloproteinase-9 (MMP-9)/Estrogen Receptor beta (ERβ) axis has been identified as a key driver of endometriosis by inhibiting apoptosis and promoting inflammatory responses within endometriotic lesions^19, 24^. Thus, we investigated whether PCB126 exposure modulates the SRC-1 isoform/MMP-9/ERβ axis in endometriotic lesions, thereby enhancing endometriosis progression. Endometriotic lesions were isolated from mice with endometriosis treated with PCB126 or vehicle control, and the levels of this regulatory axis were analyzed by Western blot. PCB126 exposure significantly increased the ratio of the SRC-1 isoform to full-length SRC-1 in endometriotic lesions compared to vehicle-treated controls (Fig. 2A). In addition, PCB126 elevated the protein levels of MMP-9 and ERβ in endometriotic lesions (Figs. 2B and 2C). These results indicate that PCB126 enhances the SRC-1 isoform/MMP-9/ERβ axis, contributing to the progression of endometriosis in this mouse model. To further validate whether PCB126 exposure also activates this axis in human endometriotic lesions, we employed immortalized human endometrial epithelial cells (IHEECs) derived from ovarian endometrioma patients^27^. PCB126 exposure increased the levels of the SRC-1 isoform, MMP-9, and ERβ in IHEECs compared to vehicle-treated controls, consistent with observations in mouse endometriotic lesions (Fig. 2D). These findings indicate that PCB126 enhances the SRC-1 isoform/MMP-9/ERβ axis in both mouse and human endometriotic lesions, thereby promoting endometriosis progression through inhibition of apoptosis and activation of inflammatory responses.

Endometriosis is an estrogen-dependent disease, with both ERβ and ERα playing critical roles in its progression^28, 29^. Therefore, it remains unclear whether PCB126 activates both estrogen receptors or selectively targets one subtype. To address this key question, HeLa cells were used, as they are widely regarded as ERα- and ERβ-negative and thus serve as an ideal model system for studying exogenous ER activation^30^. To assess ER activity, HeLa cells were transiently transfected with either an ERα or ERβ expression vector along with a luciferase reporter containing an estrogen response element (ERE). As a control, we evaluated the effect of estradiol (10 nM) on ER activity in HeLa cells. Estradiol significantly increased the transcriptional activity of both ERα and ERβ compared to vehicle-treated cells (Figs. 2E and 2F). Using this system, we next assessed whether PCB126 activates ERs. PCB126 (0.1 nM) significantly increased ERβ transcriptional activity compared to the vehicle (Fig. 2E), whereas it did not induce ERα activity (Fig. 2F). These results indicate that PCB126 preferentially activates ERβ over ERα.

To validate whether the PCB126/ERβ axis drives endometriosis progression, we employed IHEECs overexpressing ERβ (IHEECs:ERβ)^19^ and then determined whether PCB126 enhances the growth of IHEECs:ERβ compared to their parental IHEECs. The MTS cell proliferation assay revealed that PCB126 significantly increased the proliferation of IHEECs:ERβ compared to control IHEECs (Fig. 2G). These results suggest that PCB126 promotes the proliferation of IHEECs primarily through ERβ activation.

#### PCB126 activates the AXL/GAS6 axis to enhance ERβ activity in endometriotic lesions.

**Figure 3:**
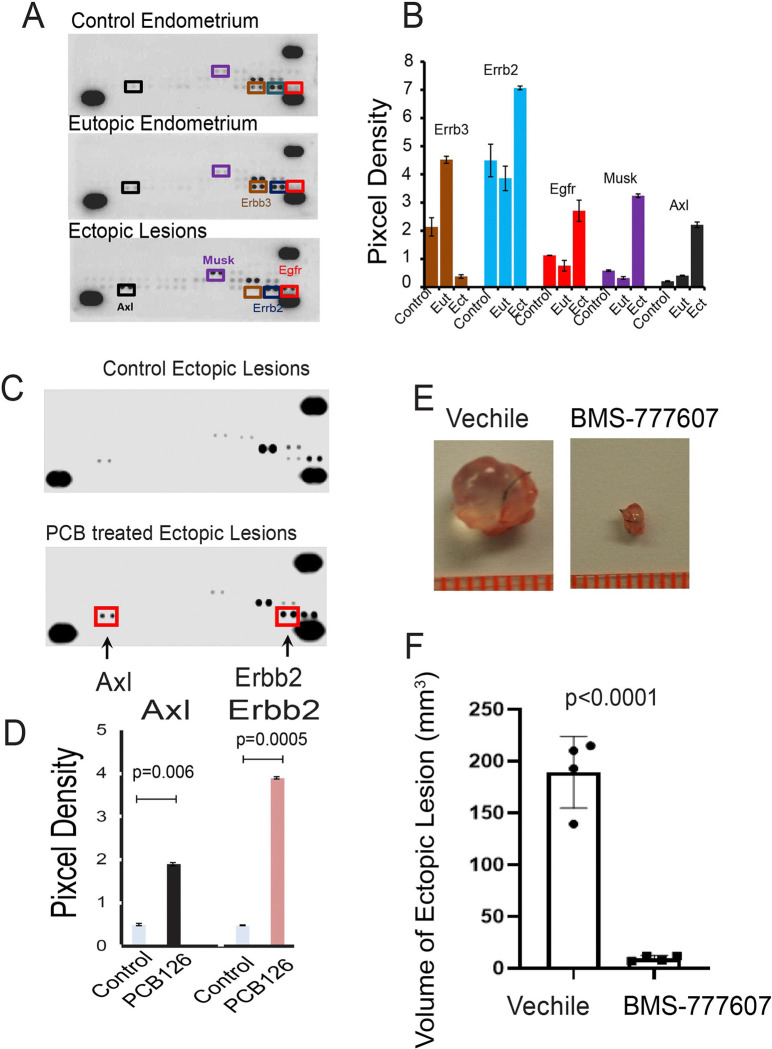
PCB126 activates AXL signaling to enhance endometriosis progression. **A–B** Analysis of receptor tyrosine kinase (RTK) activation in ectopic lesions and eutopic endometrium of mice with endometriosis, as well as control endometrium from non-endometriosis control mice (n=3 per group). Phosphorylated RTKs were detected using the Mouse Phospho-Receptor Tyrosine Kinase Array Kit (**A**). Differential levels of phosphorylated ErbB3, ErbB2, EGFR, MuSK, and AXL were quantified using the ImageJ program (**B**). Eut: eutopic endometrium; Ect: ectopic lesions. **C–D** Assessment of RTK activation in ectopic lesions of mice with endometriosis treated with PCB126 (1 mg/kg) or vehicle once weekly for 5 weeks (n=3 per group) (**C**). Phosphorylated AXL and ErbB2 levels were quantified using ImageJ (**D**). **E–F** Evaluation of the effect of the AXL inhibitor BMS-777607 on endometriosis progression. Mice with endometriosis (n=4 per group) were treated with BMS-777607 (25 mg/kg) or vehicle, five times per week for 3 weeks (**E**). The volumes of ectopic lesions from treated mice were quantified (**F**).

How does PCB126 activate ERβ in endometriotic lesions to promote endometriosis progression? Receptor tyrosine kinase (RTK) signaling has been shown to modulate ERβ activity^31, 32^ and PCB126 is known to influence RTK signaling pathways involved in regulating various cellular processes^33, 34^. RTK activation contributes to endometriosis progression and is considered a potential molecular therapeutic target for its treatment^35^. These observations suggest that ERβ activation by RTK signaling is enhanced by PCB126. To test this hypothesis, a mouse RTK array was performed using control endometrial tissue from healthy mice, as well as eutopic and ectopic endometria from mice with endometriosis (Fig. 3A). The levels of phosphorylated ErbB2 (p-ErbB2), EGFR (p-EGFR), MuSK (p-MuSK), and Axl (p-Axl) were markedly elevated in ectopic lesions compared to both eutopic and control endometria (Fig. 3B). These findings indicate that the activation of ErbB2, EGFR, MuSK, and Axl is associated with endometriosis progression in mice. The next question was which RTK(s) are specifically involved in PCB126-induced endometriosis progression. To address this, a mouse RTK array was conducted on ectopic lesions isolated from mice with endometriosis treated with either PCB126 or vehicle (Fig. 3C). PCB126 exposure significantly increased the levels of p-Axl and p-ErbB2 in endometriotic lesions (Fig. 3D). Among the RTKs associated with endometriosis, Axl and ErbB2 were preferentially activated by PCB126, suggesting their involvement in PCB126-mediated disease progression. Given that Axl has been more extensively studied in the context of endometriosis, we focused on its activation by PCB126 for further investigation^36, 37^.

Although Axl activation is associated with PCB126-induced endometriosis, direct evidence demonstrating its causal role in disease progression is lacking. To address this, we employed the Axl inhibitor BMS-777607, which has been shown to specifically reduce Axl phosphorylation, tumor invasion, and angiogenesis in glioma cells^38^. Mice with endometriosis exposed to PCB126 were treated with either BMS-777607 or vehicle as a control. BMS-777607 treatment significantly reduced the volume of endometriotic lesions in PCB126-exposed mice compared to the vehicle-treated group (Figs. 3E and 3F). These results indicate that Axl activation plays a critical role in mediating PCB126-induced endometriosis progression.

**Figure 4:**
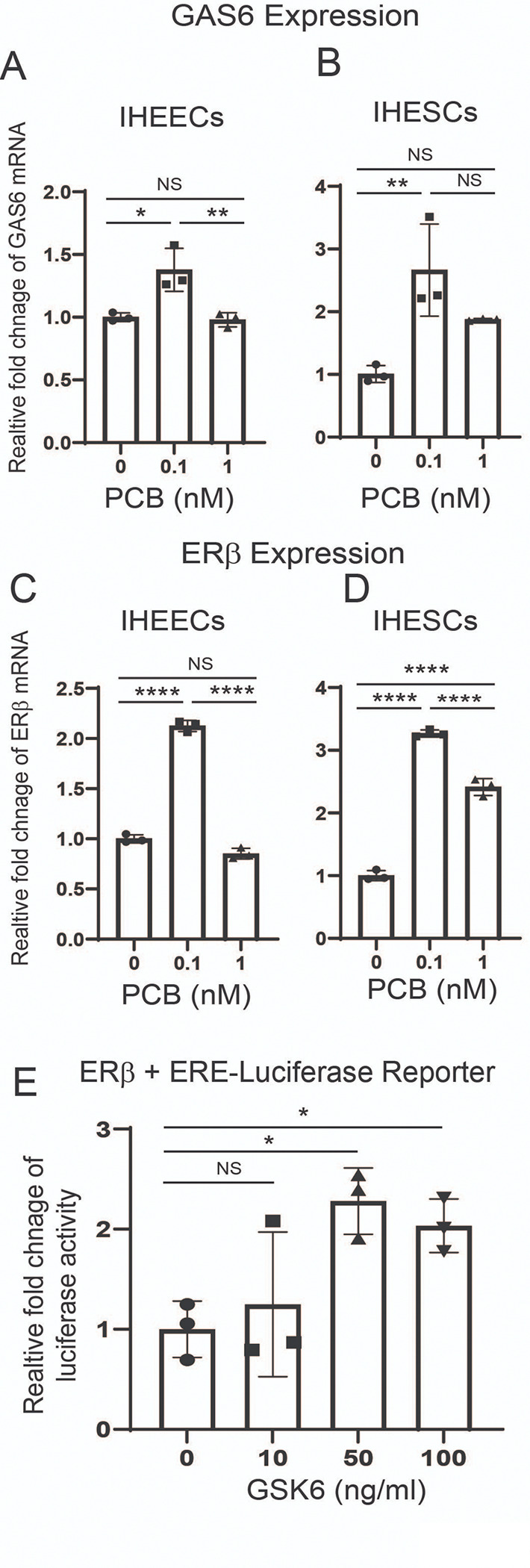
PCB126 elevated levels of GAS6/ERb axis to enhance ERbactivity **A–B** GAS6 mRNA levels in IHEECs (**A**) and IHESCs (**B**) following treatment with various doses of PCB126 or vehicle. **C–D** ERβ protein levels in IHEECs (**C**) and IHESCs (**D**) after treatment with increasing doses of PCB126 or vehicle. **E** Assessment of the intrinsic transcriptional activity of ERβ induced by GAS6. HeLa cells were transiently transfected with an ERβ expression vector and an ERE-luciferase reporter, followed by treatment with varying concentrations of GAS6 or vehicle for 48 hours. Relative luciferase activity was calculated as the fold change in GAS6-treated versus vehicle-treated cells.

The next question is how PCB126 activates Axl in endometriotic lesions. To address this, we measured the levels of GAS6, the ligand for Axl, in endometrial cells treated with PCB126 compared to vehicle. PCB126 (0.1 nM) significantly increased GAS6 mRNA levels in both IHEECs (Fig. 4A) and IHESCs (Fig. 4B). These results suggest that PCB126 upregulates GAS6 expression, leading to Axl activation in endometriotic lesions. We next asked whether PCB126 also increases ERβ levels in endometriotic lesions, thereby promoting disease progression. Similar to GAS6, PCB126 (0.1 nM) treatment elevated ERβ levels in both IHEECs (Fig. 4C) and IHESCs (Fig. 4D) compared to the vehicle. To further explore whether GAS6 can enhance ERβ activity, HeLa cells were transiently transfected with an ERβ expression vector and an ERE-luciferase reporter construct. Treatment with GAS6 (50 ng/ml) significantly increased ERβ transcriptional activity compared to vehicle-treated controls (Fig. 4E). These findings indicate that, in addition to elevating ERβ expression, PCB126 enhances ERβ activity through activation of the Axl/GAS6 signaling axis in endometriotic lesions.

#### PCB126 upregulated Dnmt3a in endometriotic lesions.

**Figure 5:**
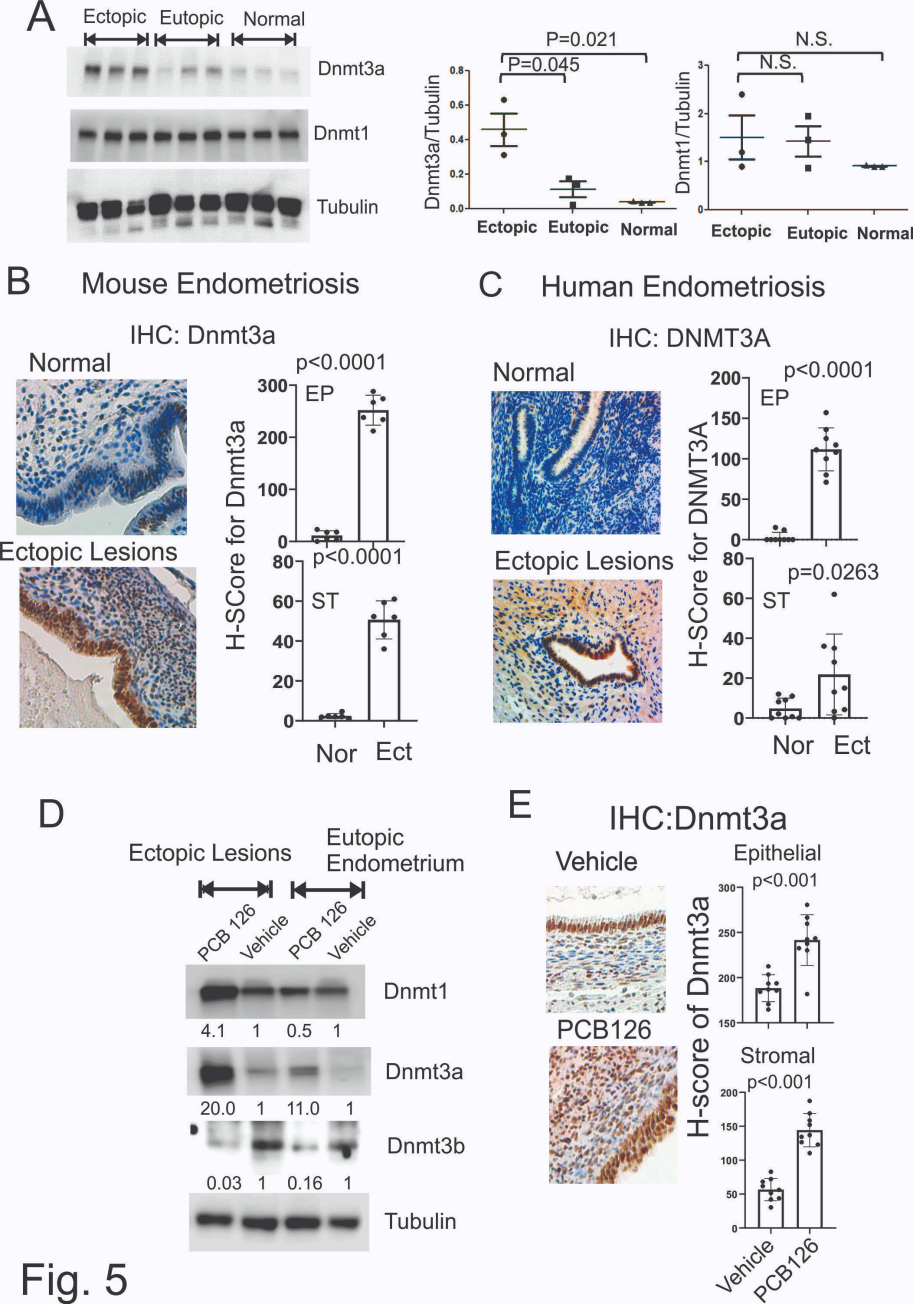
PCB126 increased the level of Dnmt3a in ectopic lesions. **A** Western blot analysis of DNMT3A, DNMT1, and tubulin (as a loading control) in ectopic lesions and eutopic endometrium of mice with endometriosis, as well as in normal endometrium from control mice without endometriosis. The DNMT3A/tubulin and DNMT1/tubulin ratios were quantified for each tissue type. **B** Immunohistochemistry (IHC) analysis of DNMT3A expression in normal endometrium from control mice and ectopic lesions from mice with endometriosis (n=6 per group). H-scores were calculated using the QuPath program. **C** IHC analysis of DNMT3A expression in normal endometrium from women without endometriosis and ectopic lesions from endometriosis patients (n=9 per group). H-scores were calculated using the QuPath program. **D** Western blot analysis of DNMT1, DNMT3A, DNMT3B, and tubulin in ectopic lesions and eutopic endometrium from mice with endometriosis treated with PCB126 (1 mg/kg) or vehicle once weekly for 5 weeks. **E** IHC analysis of DNMT3A expression in ectopic lesions from mice with endometriosis treated with PCB126 (1 mg/kg) or vehicle once weekly for 5 weeks. H-scores in epithelial and stromal compartments were quantified using the QuPath program.

Epigenetic dysregulation is associated with endometriosis progression, and PCB126 exposure has been shown to alter epigenetic states, including DNA methylation^39, 40^. Based on these findings, we investigated whether PCB126 alters the expression of DNA methyltransferases (DNMTs) in endometriotic lesions compared to control endometrium, potentially promoting endometriosis progression through lesion-specific DNA methylation. Ectopic lesions and eutopic endometrium were isolated from mice with endometriosis, along with normal uterine tissue from control mice. Western blot analysis showed that Dnmt3a levels were significantly elevated in ectopic lesions compared to both eutopic endometrium and control endometrium (Fig. 5A). In contrast, Dnmt1 levels remained unchanged across ectopic, eutopic, and normal endometrial tissues (Fig. 5A). Immunohistochemical (IHC) analysis further confirmed that Dnmt3a expression was markedly increased in both the epithelial and stromal compartments of ectopic lesions in mice with endometriosis compared to normal endometrium (Fig. 5B). To validate these findings in humans, IHC for Dnmt3a was performed on human endometriotic lesions from endometriosis patients and compared with endometrial tissue from women without the disease. Consistent with the mouse model, Dnmt3a levels were significantly elevated in both epithelial and stromal cells of human endometriotic lesions relative to normal endometrium (Fig. 5C). These results demonstrate that Dnmt3a is upregulated in ectopic lesions in both human patients and mouse models of endometriosis, suggesting a potential role in disease progression.

To determine the effect of PCB126 on Dnmt3a expression in endometriotic lesions, ectopic lesions and eutopic endometrium were isolated from mice with endometriosis treated with PCB126 or vehicle as a control. Western blot analysis showed that PCB126 treatment increased Dnmt1 levels in ectopic lesions but not in eutopic endometrium (Fig. 5D). In contrast, PCB126 significantly elevated Dnmt3a levels in both ectopic lesions and eutopic endometrium compared to vehicle-treated controls (Fig. 5D).

Conversely, PCB126 treatment reduced Dnmt3b levels in both ectopic lesions and eutopic endometrium (Fig. 5D). Furthermore, IHC analysis confirmed that PCB126 exposure markedly increased Dnmt3a expression in both epithelial and stromal cells of mouse ectopic lesions compared to vehicle-treated mice with endometriosis (Fig. 5E). These findings indicate that PCB126 exposure significantly upregulates Dnmt3a expression in endometriotic lesions in a mouse model of endometriosis.

#### Dnmt3a is an ERβ target gene in ectopic lesions.

**Figure 6:**
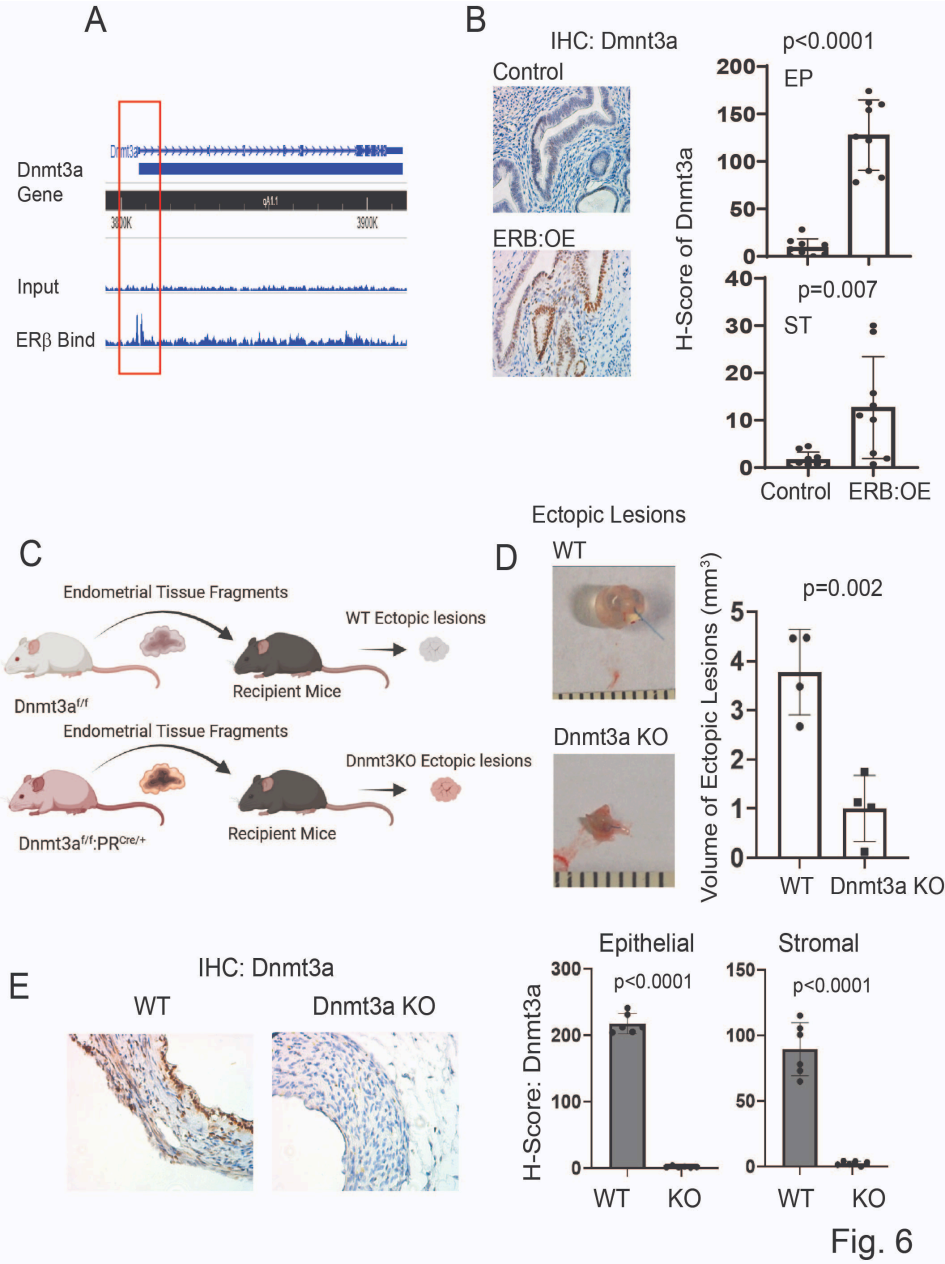
DNMT3A had a critical role in endometriosis progression. A ERβ ChIP-sequencing analysis revealed direct binding of ERβ to the promoter region of the *Dnmt3a* gene in ERβ-overexpressing ectopic lesions from mice with endometriosis. **B** Immunohistochemistry (IHC) using an ERβ antibody showed that ERβ overexpression increased DNMT3A levels in the uterus of endometrium-specific ERβ-overexpressing (ERβ:OE) mice compared to control mice. H-scores for DNMT3A in epithelial (EP) and stromal (ST) cells were quantified using the QuPath program. **C** Schematic overview of the strategy used to generate Dnmt3a knockout (KO) and wild-type (WT) ectopic lesions in syngeneic recipient mice. Endometrial fragments were obtained from the uteri of Dnmt3a^f/f^ :PR^Cre/+^ mice for the KO group and from Dnmt3a^f/f^ mice for the WT control group. **D** Volume analysis of Dnmt3a KO versus WT ectopic lesions. Lesions were harvested from recipient mice on day 21 after endometriosis induction. **E** Evaluation of DNMT3A expression in Dnmt3a KO and WT ectopic lesions (from panel D). H-scores for DNMT3A in epithelial and stromal cells of KO and WT lesions were determined using the QuPath program.

How is Dnmt3a upregulated in endometriotic lesions? This is a key question for understanding Dnmt3a-mediated endometriosis progression. To investigate this, we reanalyzed a previously published ERβ chromatin immunoprecipitation sequencing (ChIP-seq) dataset specific to endometriotic lesions^29^ and found that ERβ directly binds to the promoter region of the *Dnmt3a* gene (Fig. 6A). To determine whether ERβ binding increases Dnmt3a expression in endometriotic lesions, we utilized our ERβ overexpression mouse model (ROSA^LSL:ERβ/+^:Progesterone Receptor (PR)^Cre/+^, ERB:OE) in which ERβ levels are significantly elevated across all compartments of the uterine tissue^19^. IHC analysis for Dnmt3a revealed markedly elevated expression in both epithelial and stromal cells of the uterus in ERβ:OE mice compared to control mice (PR^Cre/+^) (Fig. 6B). These results indicate that ERβ functions as a key transcriptional regulator that directly upregulates Dnmt3a expression in the uterine endometrium.

#### Dnmt3a has a critical role in endometriosis progression.

To determine whether Dnmt3a plays an essential role in endometriosis progression, we generated an endometrium-specific Dnmt3a knockout (KO) mouse by crossing floxed Dnmt3a ((Dnmt3a^f/f^)^21^ with Progesterone Receptor (PR)^Cre/+^ mice, in which Cre recombinase is expressed in PR-expressing cells^20^. To generate Dnmt3a knockout (KO) ectopic lesions, uteri were isolated from Dnmt3a^f/f^:PR^Cre/+^ female mice, and endometrial fragments were implanted into syngeneic female recipient mice (Fig. 6C). As a control, uteri were isolated from Dnmt3a ^f/f^ female mice, and control endometrial fragments were similarly implanted into syngeneic recipients (Fig. 6C). Comparative analysis revealed that the volume of Dnmt3a KO ectopic lesions was significantly smaller than that of control lesions (Fig. 6D). To validate Dnmt3a deletion in the ectopic lesions, IHC with a Dnmt3a-specific antibody was performed on both KO and control lesions. The staining confirmed that Dnmt3a expression was absent in both epithelial and stromal cells of Dnmt3a KO lesions, while it was readily detected in control lesions (Fig. 6E). Collectively, these results demonstrate that Dnmt3a plays a critical role in the progression of endometriosis in this mouse model.

#### Dnmt3a regulates inflammatory immune response in ectopic lesions for the progression of endometriosis.

**Figure 7:**
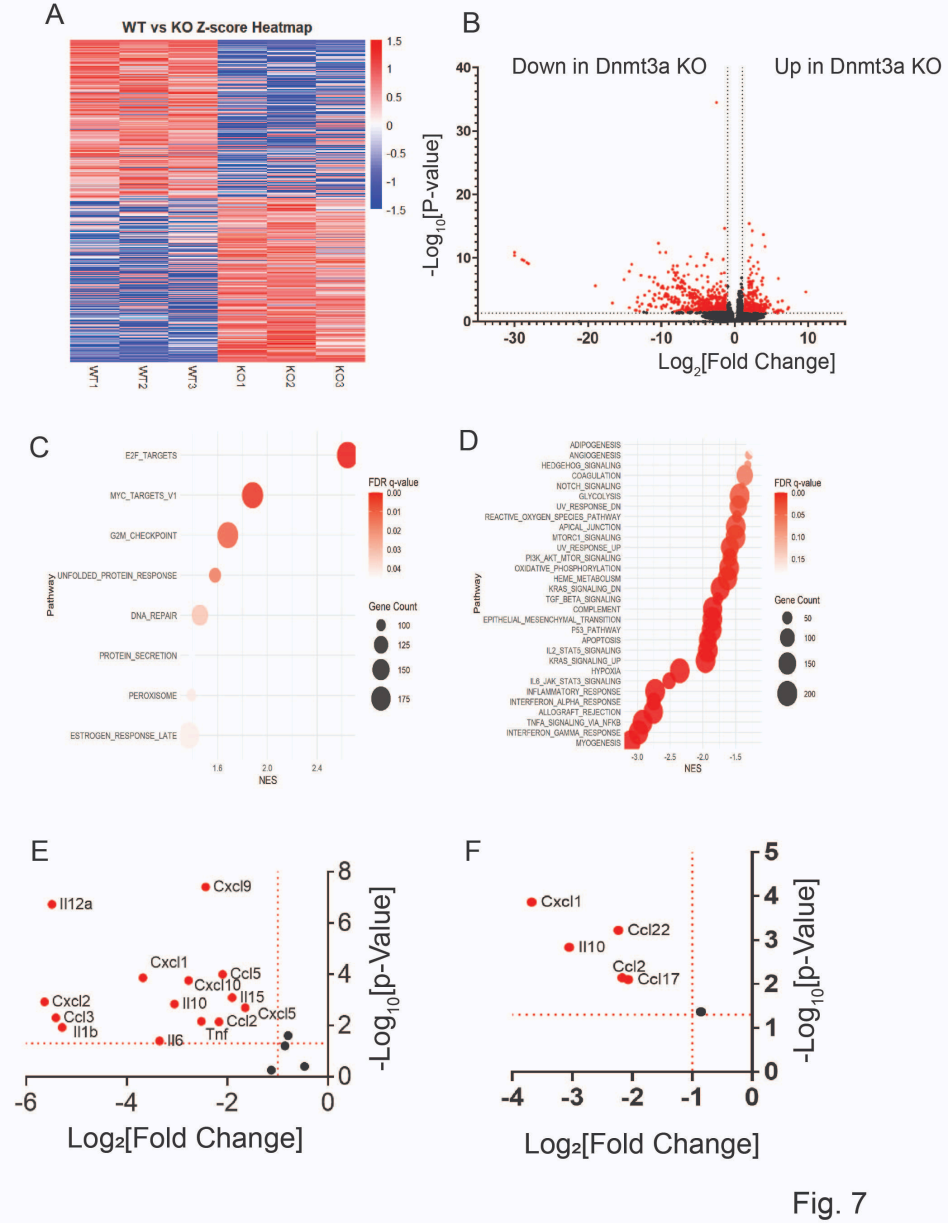
DNMT3A has a critical role in cytokine-mediated immune response in ectopic lesions in mice withendometriosis. **A** Z-score heatmap analysis of Dnmt3a knockout (KO, n=3) and wild-type (WT, n=3) ectopic lesions from recipient mice with endometriosis. **B** Volcano plot showing differential gene expression in *Dnmt3a* KO versus WT ectopic lesions. Genes with significant changes (log₂[Fold Change] ≥ ±1, −log₁₀[P-value] > 1.3) are highlighted in red. **C–D**Gene Set Enrichment Analysis (GSEA) with bubble plots illustrating gene ontology terms enriched in upregulated (**C**) and downregulated (**D**) genes in *Dnmt3a* KO ectopic lesions. Analyses were based on normalized enrichment score (NES), false discovery rate (FDR), and gene counts. **E**Expression levels of cytokines and chemokines previously implicated in endometriosis progression. **F** Expression levels of cytokines and chemokines known to contribute to an immunosuppressive microenvironment by recruiting regulatory T cells (Tregs) and myeloid-derived suppressor cells (MDSCs).

How does Dnmt3a drive endometriosis progression? To address this key question, RNA expression profiles of control ectopic lesions (n = 3) and *Dnmt3a* knockout (KO) ectopic lesions (n = 3) were analyzed using bulk RNA-seq. A heatmap of Z-scores revealed distinct RNA expression patterns between *Dnmt3a* KO and control ectopic lesions (Fig. 7A). Differential gene expression analysis identified 251 genes significantly upregulated in *Dnmt3a* KO ectopic lesions (− log₁₀[p-value] > 1.3 and log₂[fold change] > ± 1.0). In contrast, a substantially larger number of genes—708 in total—were significantly downregulated in *Dnmt3a* KO lesions compared to controls (− log₁₀[p-value] > 1.3 and log₂[fold change] > ± 1.0) (Fig. 7B). Gene Set Enrichment Analysis (GSEA) showed that only a few cellular pathways, including E2F targets, MYC targets, and the G2/M checkpoint, were upregulated in *Dnmt3a* KO lesions relative to controls (Fig. 7C). In contrast, many cellular pathways were significantly downregulated in *Dnmt3a* KO lesions (Fig. 7D). Notably, most of these downregulated pathways—including angiogenesis, reactive oxygen species (ROS) signaling, epithelial–mesenchymal transition (EMT), PI3K-AKT signaling, and inflammatory signaling—are well-established as essential contributors to endometriosis progression^41, 42^. Among these, inflammatory response-related pathways—including interferon, interleukin, TNFα, and TGFβ signaling—were markedly and significantly downregulated in *Dnmt3a* KO ectopic lesions compared to control ectopic lesions (Fig. 7D). To validate this observation, we selected cytokines and chemokines known to play critical roles in endometriosis^43, 44, 45^. Most of these cytokines and chemokines were significantly reduced in Dnmt3a KO ectopic lesions compared to control ectopic lesions (Fig. 7E). These findings suggest that Dnmt3a is a key driver of cytokine and chemokine expression associated with endometriosis. Endometriotic lesions establish a profoundly immunosuppressive microenvironment that supports ectopic tissue survival and progression. Consequently, regulatory T cells, myeloid-derived suppressor cells (MDSCs), and M2 macrophages are highly enriched within ectopic lesions^46, 47, 48^. Based on these observations, we measured the expression levels of *Cxcl1, Ccl22, Ccl2, Ccl17,* and *Il10*, as these chemokines are involved in the recruitment of immunosuppressive cells^49, 50^. The levels of these chemokines were significantly reduced in Dnmt3a KO endometriotic lesions compared to control ectopic lesions (Fig. 7F). These results indicate that Dnmt3a plays a critical role in establishing the immunosuppressive microenvironment of ectopic lesions, thereby promoting endometriosis progression.

### Discussion

Several hypotheses have been proposed to explain how endometriosis is initiated and progresses^51^. However, the causal factors underlying the initiation of endometriosis remain poorly defined. Exposure to endocrine-disrupting chemicals (EDCs), such as PCBs, TCDD, BPA, and their analogs, is strongly associated with the progression of endometriosis by activating multiple intracellular signaling pathways, including those related to inflammation, estrogen and progesterone signaling, cell survival, and apoptosis^3^. Based on these observations, EDCs are considered potential causal factors in the progression of endometriosis. In addition to EDCs, estrogen is recognized as a key driver of endometriosis progression^52^. Therefore, synergistic interaction between estrogen and EDC signaling is likely critical for the progression of endometriosis. How EDCs coordinate with estrogen signaling in endometriotic lesions to promote disease progression remains unclear. A previous study demonstrated that PCB126 can directly induce transcriptional activation of ERα and estrogenic responses in the absence of ER agonists, owing to its intrinsic estrogenic activity^53^. However, there is no direct evidence demonstrating the agonistic activity of PCB126 toward ER. In this context, the PCB126/AXL/ERβ axis may provide a critical clue to understanding how PCB126 coordinates with ERβ to promote endometriosis progression.

How does PCB126 activate ERβ in endometriotic lesions to promote disease progression? In this study, we demonstrate that PCB126 enhances ERβ activity through the activation of AXL receptor tyrosine kinase. AXL activation is closely associated with endometriosis progression. For instance, the expression of growth arrest-specific gene 6 (*GAS6*), the ligand for AXL, and *AXL* mRNA levels are significantly elevated in endometriotic endometrial tissue compared to normal endometrium^36^. Therefore, the GAS6–AXL signaling pathway is believed to be aberrantly activated in endometriotic lesions, contributing to disease progression by enhancing ERβ activity, as GAS6 has been shown to stimulate the intrinsic transcriptional activity of ERβ. The next critical question is how the AXL/GAS6 axis activates ERβ in endometriotic lesions. Unfortunately, there is currently no direct evidence addressing this mechanism. Notably, activation of the AXL/GAS6 axis initiates several downstream signaling pathways, including PI3K–AKT–mTOR, MEK–ERK, NF-κB, and JAK/STAT^54^. Activation of these kinase signaling pathways is strongly associated with endometriosis progression^55, 56, 57, 58^. Moreover, these pathways have been shown to enhance the transcriptional activity of ERβ^59, 60^ Therefore, we propose that kinases activated by the PCB126/AXL/GAS6 axis may, in turn, activate ERβ to promote endometriosis progression. Further studies are needed to identify which AXL downstream kinase specifically activates ERβ in endometriotic lesions.

Alterations in epigenetic regulation are strongly associated with the progression of endometriosis^61^. In patients with endometriosis, DNMT1, DNMT3A, and DNMT3B are overexpressed in the epithelial component of endometriotic implants compared to normal controls or the eutopic endometrium of women with endometriosis^16, 61^. However, other studies have reported significantly lower expression levels of DNMTs in endometriotic lesions compared to the eutopic endometrium of women with endometriosis and to disease-free controls^17^. There are controversial studies regarding the differential expression of DNMTs in endometriotic lesions compared to normal endometrium. Therefore, the expression profile of DNMTs in endometriosis may be context-dependent. Our study revealed that DNMT3A levels are elevated in both mice and human endometriotic lesions. Identifying the causal factor responsible for DNMT3A upregulation in endometriotic lesions is a key question for understanding the molecular etiology of endometriosis progression. However, this question remains unresolved. Here, we propose that exposure to PCB126 drives the upregulation of DNMT3A, leading to the establishment of endometriosis-associated DNA methylation patterns that promote disease progression. Notably, PCB126 exposure alters global DNA methylation levels in a context-dependent manner. For example, in elderly Swedish individuals, elevated levels of PCB126 were associated with global DNA hypermethylation^62^. However, some studies have linked PCB exposure to global DNA hypomethylation^63^. These global methylation changes could be linked to the dysregulation of DNMT expression induced by PCB126. For example, PCB exposure significantly increased DNMT3A and DNMT3B expression in the Leydig cells of progeny rats, impairing testosterone production^64^. In contrast, a mixture of PCBs—including PCB126—reduced the expression of DNMT1, DNMT3A, and DNMT3B in the livers of female offspring^40^. Therefore, PCB exposure alters DNMT levels in a context-dependent manner. Our study demonstrated that PCB126 elevates DNMT3A expression in endometriotic lesions, contributing to the establishment of endometriosis-associated DNA methylation.

How is DNMT3A upregulated in endometriotic lesions? Answering this question is critical to understanding DNMT3A-mediated endometriosis progression. Activating Protein 2 alpha (AP2A) and Octamer-binding Transcription Factor 1 (OCT1) have been shown to transactivate DNMT3A expression^65, 66^. However, EndometDB analysis revealed that these transcription factors are not upregulated in endometriotic lesions compared to normal endometrium^67^. Our study identified ERβ as a causal factor that enhances DNMT3A expression in endometriotic lesions. ERβ is a well-established driver of endometriosis, and PCB126 enhances ERβ activity through the AXL/GAS6 signaling axis. Therefore, the PCB126/AXL/GAS6/ERβ axis represents a key mechanism underlying the induction of endometriosis-associated DNA methylation via DNMT3A. This provides an important insight into how PCB126 exposure alters DNA methylation to promote endometriosis progression.

**Figure 8:**
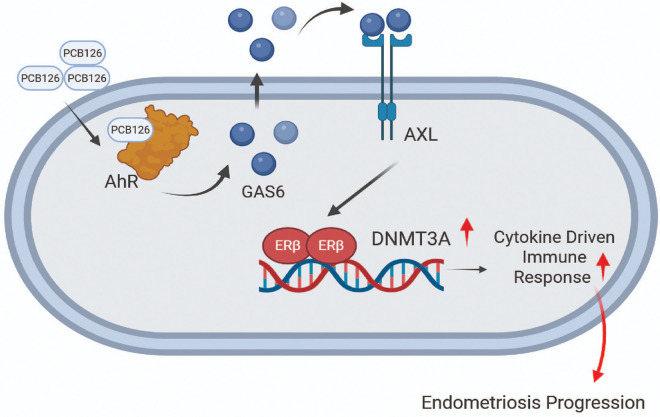
The AXL/GAS6/ERβ/DNMT3A axis in PCB126-induced endometriosis progression. PCB126 exposure elevated GAS6 levels in endometriotic lesions, leading to the activation of AXL signaling. Activated AXL enhanced ERβ activity, which in turn upregulated DNMT3A expression. Elevated DNMT3A contributed to the development of endometriosis-associated inflammatory and immune microenvironment, promoting disease progression.

Although elevated DNMT3A levels are associated with endometriosis progression, there is no direct evidence demonstrating that alterations in DNMT3A drive disease progression. In this context, our endometrium-specific Dnmt3a knockout mouse model provides critical insight, showing that DNMT3A plays a causal role in endometriosis progression. This raises the question: what is the functional role of DNMT3A in this process? A previous study reported that 15.4% of the variation in endometriosis is attributable to DNA methylation and identified significant differences in DNA methylation profiles associated with stage III/IV endometriosis^68^. Gene ontology analysis of differentially methylated genes revealed enrichment in pathways related to cellular proliferation, neuronal function, extracellular matrix–cell interactions, and cancer-associated signaling, including MAPK, Wnt, calcium, and Hippo pathways, as well as focal adhesion, axon guidance, and pathways implicated in breast and gastric cancers^68^. These pathways are known to play critical roles in endometriosis progression. However, there is no direct evidence linking these endometriosis-associated DNA methylation changes to alterations in DNMTs. Our Dnmt3a knockout mouse model demonstrated that many of these cellular pathways were downregulated in ectopic lesions lacking Dnmt3a, suggesting that DNMT3A is involved in modulating key endometriosis-associated pathways. Beyond these cellular mechanisms, our RNA-seq analysis further revealed that Dnmt3a plays a critical role in shaping the inflammatory and immune microenvironment of ectopic lesions. Several studies have also shown that altered cytokine and chemokine expression, along with differential immune cell recruitment, are strongly associated with endometriosis progression^69^. However, the mechanisms underlying the distinct inflammatory and immune environment within ectopic lesions remain poorly understood. The PCB126/AXL/GAS6/ERβ/DNMT3A axis provides a critical clue for understanding how EDCs may epigenetically drive the formation of an inflammatory-immune microenvironment that promotes endometriosis progression (Fig. 8). Collectively, our study identifies the PCB126/AXL/GAS6/ERβ/DNMT3A axis as a key integrator of endocrine-disrupting chemical (EDC)-mediated endometriosis progression.
